# Accelerated orthodontic tooth movement: surgical techniques and the regional acceleratory phenomenon

**DOI:** 10.1186/s40902-021-00331-5

**Published:** 2022-01-05

**Authors:** Elif Keser, Farhad B. Naini

**Affiliations:** 1 Department of Orthodontics, Boston University Henry M. Goldman School of Dental Medicine, Boston, USA; 2 Kingston and St George’s Hospitals and St George’s Medical School, Blackshaw Road, London, SW17 0QT UK

**Keywords:** Regional acceleratory phenomenon, Microosteoperforations, Periodontally accelerated osteogenic orthodontics (PAOO), Corticision, Piezocision

## Abstract

**Abstract:**

**Background:**

Techniques to accelerate tooth movement have been a topic of interest in orthodontics over the past decade. As orthodontic treatment time is linked to potential detrimental effects, such as increased decalcification, dental caries, root resorption, and gingival inflammation, the possibility of reducing treatment time in orthodontics may provide multiple benefits to the patient. Another reason for the surge in interest in accelerated tooth movement has been the increased interest in adult orthodontics.

**Review:**

This review summarizes the different methods for surgical acceleration of orthodontic tooth movement. It also describes the advantages and limitations of these techniques, including guidance for future investigations.

**Conclusions:**

Optimization of the described techniques is still required, but some of the techniques appear to offer the potential for accelerating orthodontic tooth movement and improving outcomes in well-selected cases.

## Background

The New York Times published an article in February 2012 indicating a 58% increase in the number of American adults getting orthodontic treatment from 1994 to 2010, whereas there was only a 15% increase in the number of patients under 18 years of age, during the same period. There has been a gradual increase in the number of adults starting orthodontic treatment in the USA, based on the results of orthodontic practice surveys [1]. The number of adults seeking orthodontic treatment appears to be on the increase in the UK as well [2]. A survey of the American Association of Orthodontists (AAO) showed that the number of adult patients increased by 16% in the period from 2012 to 2014, and a 2013 study conducted for the AAO stated that adults who had orthodontic treatment by an orthodontist had reported significant life improvements, both personally and professionally, with 75% reporting improvements in personal relationships or in their career, which they felt was due to their improved smiles. Ninety-two percent of the adults surveyed said they would recommend orthodontic treatment to other adults [3]. A study conducted at the Eastman Dental Institute in the UK found that the desire for dental alignment and improved smile aesthetics were the primary motivating factors for initiating treatment [4]. There are significant differences between the orthodontic treatment of adolescents and adults. These are mainly related to psychological and biological differences.

Tayer [5] found that 33% of adult patients were discouraged from having orthodontic treatment due to the length of treatment and the discomfort and inconvenience involved with having orthodontic appliances. A major concern for adult orthodontic patients is the prolonged length of treatment. Adults tend to desire a shorter treatment time and demand more aesthetic appliances [6, 7].

The demand for more aesthetic appliances has been addressed by the use of clear aligners and lingual orthodontics. In the past decade, various techniques have been described to accelerate tooth movement, such as photobiomudulation, use of ultrasonic vibration, pharmacological approaches, ultrasounds, microosteoperforations (MOPs), periodontally accelerated osteogenic orthodontics **(**PAOO), corticision, and piezocision. This article is a comprehensive narrative review, which aims to summarize the surgical methods to accelerate orthodontic tooth movement and review the main studies published in recent years on this topic.

## Search strategy

The search strategy comprised the following: The Medline, Scopus, Embase, and Web of Science databases were searched from 1985 to 2021 to identify all studies using a number of terms. These included regional acceleratory phenomenon, RAP, accelerated orthodontics, microosteoperforations, micro-osteoperforations, periodontally accelerated osteogenic orthodontics **(**PAOO), corticision, and piezocision. The Boolean operators “and” and “not” were used to focus the search. The following journals were also hand-searched from 2000 to 2021:

*American Journal of Orthodontics (and subsequent* *American Journal of Orthodontics and Dentofacial* *Orthopedics)*

*Angle Orthodontist*

*British Journal of Orthodontics (and subsequent Journal of Orthodontics)*

*European Journal of Orthodontics*

*International Journal of Oral and Maxillofacial Surgery*

*International Journal of Periodontics and Restorative Dentistry*

*Journal of Clinical Orthodontics*

*Journal of Oral and Maxillofacial Surgery*

*Journal of Periodontology*

*Seminars in Orthodontics*

## Historical development of techniques

### Corticotomy

The idea behind using surgery to accelerate tooth movement is not new. The origin of surgically accelerated tooth movement dates back towards the end of the nineteenth century, having been modified during the twentieth century.

L.C. Bryan first described surgically assisted orthodontic tooth movement in 1893 [8]. Later, in 1931, Bichlmayr [9] described a surgical procedure to accelerate the correction of severe maxillary protrusion and reduce relapse, for patients over 16 years of age. The technique consisted of removing wedges of bone in order to reduce the bone volume through which root movement would occur.

The first English language paper describing a partial decortication of the alveolar bone to facilitate orthodontic tooth movement was by Köle [10]. This formed the basis of the techniques employed today. This was due to the rationale that the primary resistance to tooth movement was from the bone cortex. This cortical layer takes longer to remodel than the trabecula of the spongiosa, and if its continuity is disrupted, orthodontic tooth movement can be completed much faster. Köle reflected full-thickness flaps to expose the lingual and buccal alveolar bone. He then made interdental cuts through the cortical bone, but only slightly penetrating the medullary bone. Subapical horizontal cuts were undertaken as osteotomies, connecting the interdental cuts, and penetrating the full-thickness of the alveolus. This created a block of bone, embedding one or more teeth, and separated from the rest of the alveolar bone by the cortical osteotomy. This was referred to as a “bony block movement”.

In 1978, Generson et al. [11] published two cases using corticotomy with orthodontic treatment, stating that there was a reduction in the treatment time. Gantes et al. [12] used Köle’s technique and the removal of cortices adjacent to an extraction site in five patients with different malocclusion. The mean overall treatment times were 14.8 months in the corticotomy group and 28.3 months in the control group. The author analysed probing depths, plaque scores, and clinical attachment levels, concluding that this surgery caused minimal changes in the periodontium. Suya [13] refined Köle’s work and used “corticotomy-facilitated orthodontic” surgical treatments on 395 patients. He used a supra-apical horizontal corticotomy cut instead of the horizontal osteotomy used in Köle’s procedure. The cases took 6 to 12 months to complete. He observed that this procedure was less painful and produced less root resorption and relapse. He recommended completing the tooth movement in 3–4 months, as after this time, fusion of the edges of the blocks of bone would begin.

### Dental distraction (dentoalveolar distraction osteogenesis)

**Fig. 1:**
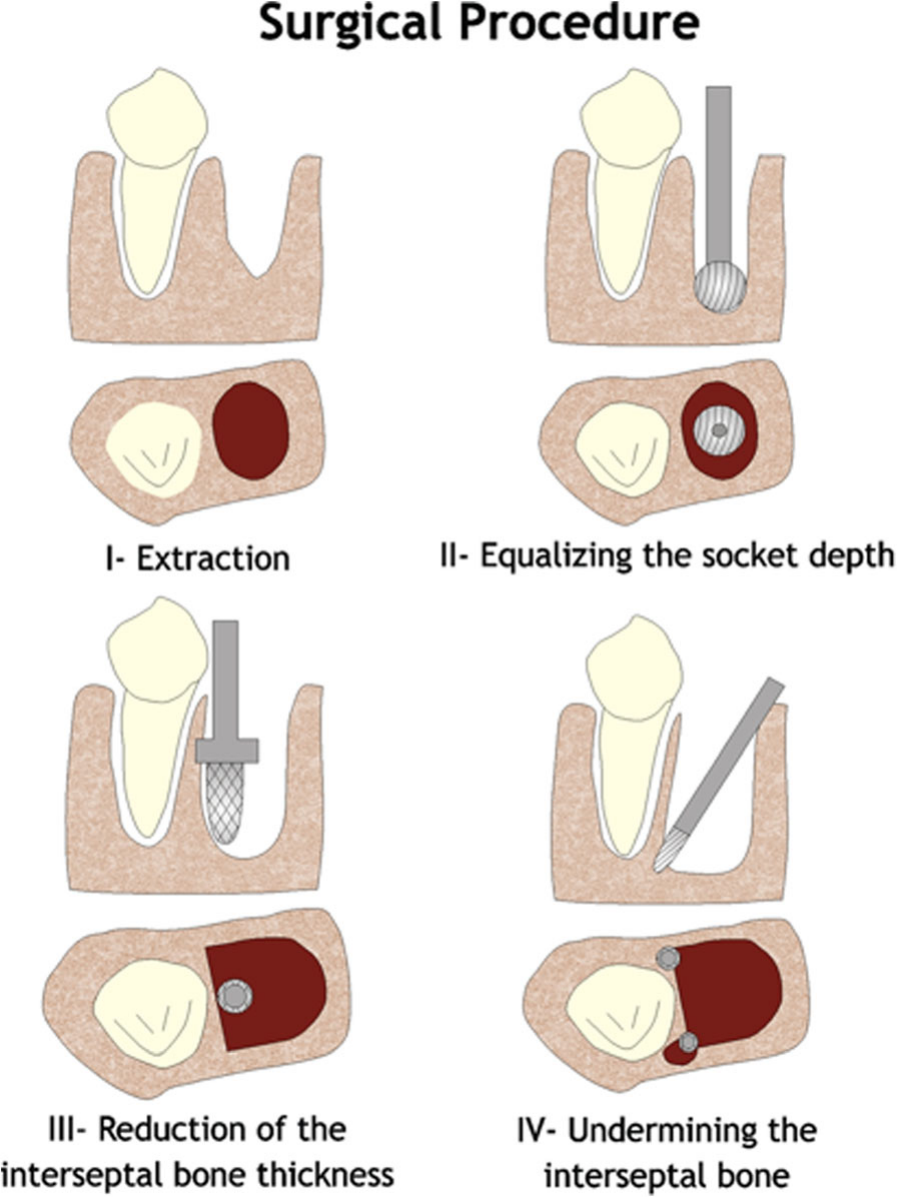
Schematic representation of the surgical procedure for rapid canine distraction (redrawn from Liou, 1998)

**Fig. 2:**
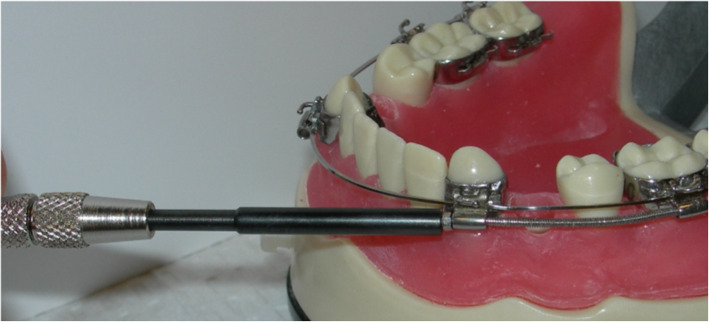
A rapid canine distraction device was designed by Drs. Keser and Pober (patent pending) to overcome some of the disadvantages of the dental distractors

**Fig. 3:**
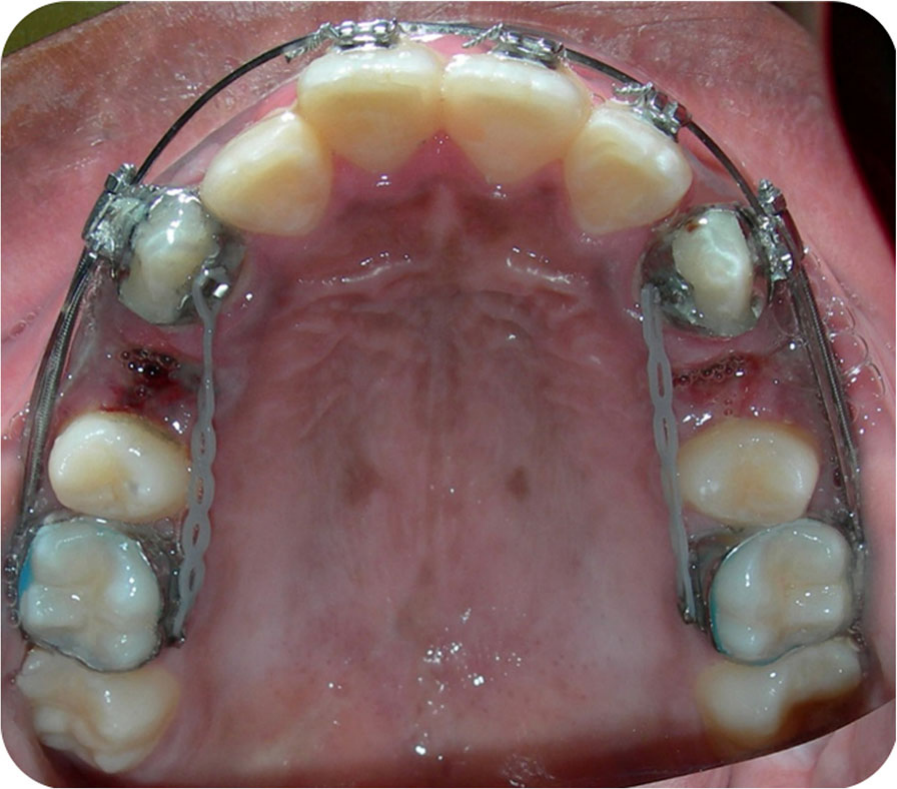
Application of the Canine distractor. Activation begins immediately after the surgical procedure and activation is at a rate of 0.35 mm twice per day. Power chain is placed on the lingual aspect to prevent rotation during the movement

Liou and Huang [14] presented a concept of “distracting the periodontal ligament” to shorten the canine distalization time to 3 weeks and called this concept “dental distraction”. This technique basically required modification of the extraction socket by undermining the interseptal bone, distal to the canines, and using an intraoral distraction device, which was activated 0.5–1 mm/day immediately after the extraction. The rapid canine retraction techniques require the use of a rigid distraction device that can be activated 0.5–1 mm/day to move the canines distally (Figs. 1, 2, and 3).

### The regional acceleratory phenomenon

Regional acceleratory phenomenon (RAP) was defined by Frost [15] as a “complex reaction of mammalian tissues to diverse noxious stimuli”. He stated that “The phenomenon occurs regionally in the anatomical sense, involves both soft and hard tissues, and is characterized by an acceleration and domination of most ongoing normal vital tissue processes. It may represent an “SOS” mechanism which evolved to potentiate tissue healing and local tissue defensive reactions.” The intensity of its response and the size of the affected region varies directly with the magnitude of the stimulus, although there is individual variability. The effect on the bone is a reduction in regional bone density due to an increased remodelling space. Frost, who was an orthopaedic surgeon, also stated that a joint contracture, which was previously too rigid to respond to traction, can respond more readily during the 3 months or so following a major local bone operation, a phenomenon which suggests that the RAP rendered the capsular and related tissues more plastic for a time.

Bogoch [16] used a rabbit model to investigate the RAP. An incomplete osteotomy of the condyle was undertaken, and calcein was included in the diet to mark the newly formed bone. At 4 weeks, he recorded newly formed cancellous bone that obliterated the osteotomy gap, with a fivefold increase in new bone, without a change in bone volume. This was given an explanation of a local acceleration in bone remodelling.

## RAP and tooth movement: a physiological concept

### Periodontally accelerated osteogenic orthodontics

Yaffe et al. [17] were the first authors who described RAP applied to the mandible, in 1994. They explored whether RAP occurs following mucoperiosteal flap surgery in the jaw bone. They found that, in rats, the elevation of a mucoperiosteal flap is sufficient to create RAP. The RAP was observed as early as 10 days after surgery, peaked at 3 weeks, and was essentially completed by or following 120 days. Both on the lingual and buccal aspects, the resorption was more prominent when a mucoperiosteal flap had been undertaken.

**Fig. 4:**
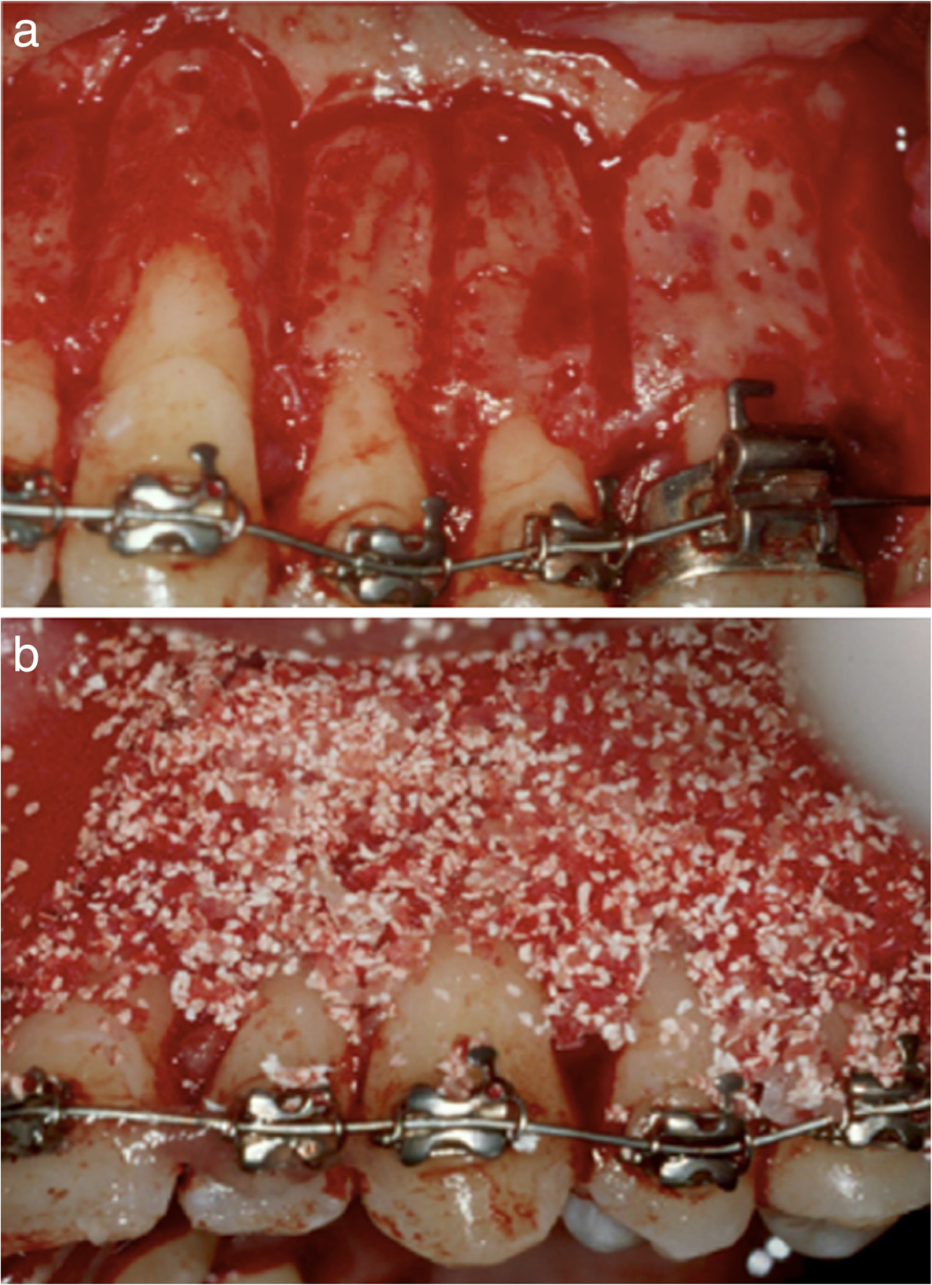
Wilcko and Wilcko PAOO. **a** Corticotomies, vertical lines and dots. **b** DFDBA placement

In 2001, Wilcko and Wilcko [18] explained the acceleration of the tooth movement in a different way. They concluded that the acceleration was due to the RAP. They changed the understanding from the mechanical concept of a “bony block movement” relating the corticotomy effect, to a physiological concept, the RAP (decalcification/recalcification of the alveolus), which was described by Frost. The Wilcko brothers introduced the Periodontally Accelerated Osteogenic Orthodontics (PAOO) technique, in which orthodontic forces were applied 1 week before the procedure, and also added bone grafts after decortication. Both on the buccal and lingual sides of the selected area, intrasulcular incisions were made, a full-thickness flap was reflected and the corticotomies were performed. The vertical incisions were located between the teeth and connected apically, and additional corticotomies were performed. Demineralized freeze-dried bone allograft (DFDBA) and bovine graft were placed and the flap was sutured (Fig. 4). The drawbacks of this technique are:

1. The length of the procedure: the procedure on both jaws can take 3–4 h to complete.
2. Due to the extent of the surgery postoperative complications are common, such as swelling, bruising, and pain.
3. Appearance of interproximal black triangles.
4. Due to the extent of the surgery, its clinical and patient acceptance is low.

### Corticision

Kim et al. [19] introduced the corticision technique as a minimally invasive alternative to create surgical injury to the bone without raising a flap. Kim et al. [19] and Park [20] use a reinforced scalpel and a mallet to go through the gingiva and the cortical bone, without flap reflection. The surgical injury created in this manner is enough to induce the RAP effect and accelerate the orthodontic tooth movement. The advantage of this technique is the avoidance of a surgical flap, when compared with the PAOO surgery. The drawbacks of this technique are:

1. The inability to graft hard or soft tissues during the procedure in order to correct and reinforce the periodontium.
2. The repeated malletting which may cause dizziness after the surgery.
3. The possibility of breakages of the scalpel and risk of injuries.

### Piezocision

Practitioners and patients often consider corticotomies to be too invasive, therefore, their acceptance remains low [21]. A new minimally invasive surgical approach without flap elevation, called piezocision, was developed to overcome this problem. First described in 2009 by Dibart [22], it combined the flapless approach of corticision with the advantage of grafting offered by PAOO. This technique combines buccal gingival micro-incisions that allow the use of the piezoelectric knife to decorticate the alveolar bone and thereby initiate the regional accelerator phenomenon. The technique is minimally invasive, but allows hard tissue and/or soft tissue grafting via selective tunnelling, in order to correct gingival recessions or bone deficiencies.

**Fig. 5:**
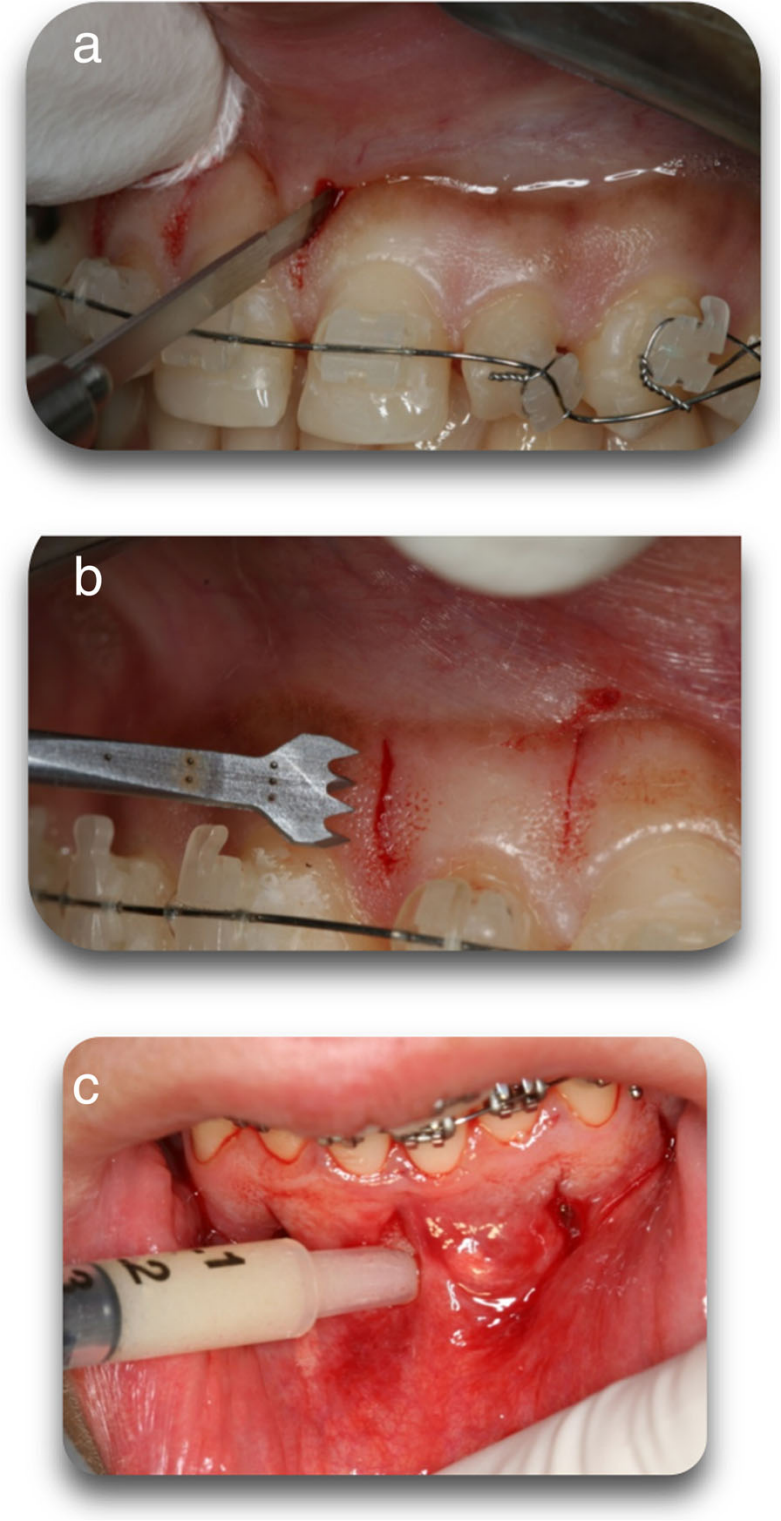
**a** Minimal vertical interproximal incisions on the buccal aspect to provide access to the piezosurgical knife. **b** Corticotomies are performed with a Piezoelectrical surgical knife (BS1 insert, Piezotome, Satelec Acteon Group, Merignac, France) through the gingival incision in the bone, with a depth of 3 mm to pass the cortical plate. **c** Where bone grafting is needed a small periosteal elevator is used to create a tunnel which will accommodate the bone graft

Piezoelectric surgery utilizes ultrasonic microvibrations to ensure that only brittle mineralized tissue is cut and soft tissues are spared, since cutting soft tissue requires a frequency above 50 kHz. Because of its micrometrical and selective cut, a piezoelectric device allows safe and precise osteotomies, without any osteonecrotic damage [23, 24]. Piezoelectric surgery also obviates the need for excessive force (Fig. 5a–c). Suturing is recommended in all areas nowadays to limit scarring. There is minimal patient discomfort. Other advantages of the procedure are the grafting option and limited duration of surgery. There is no need for flap elevation, and therefore the surgical time is decreased, and the postoperative discomfort is minimal. The drawback of this technique is that since there is no flap reflection, the cuts are undertaken blindly. The use of navigation or surgical stents is considered to be beneficial to avoid injuries to the roots.

### Micro-osteoperforations

**Fig. 6:**
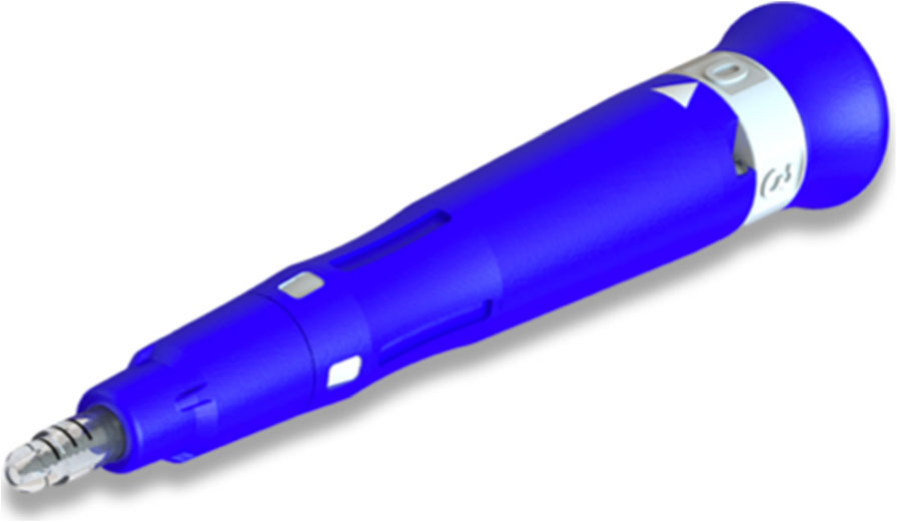
Propel device by Propel Orthodontics, USA. It has a surgical tip 1.6 mm in diameter and a usable length of up to 7.0 mm

Also known as alveocentesis, micro-osteoperforations is a novel technique introduced to accelerate tooth movement, with minimal surgical intervention. It is a minimally invasive procedure, as there is no flap elevation or incisions prior to the osteoperforations. Propel (Propel Orthodontics, USA) is an example of a device that can be used to create MOPs (Fig. 6). The effectiveness of the technique in accelerating tooth movement is theoretically the amplification in the expression of inflammatory markers that are normally expressed during orthodontic tooth movement [25, 26].

**Fig. 7:**
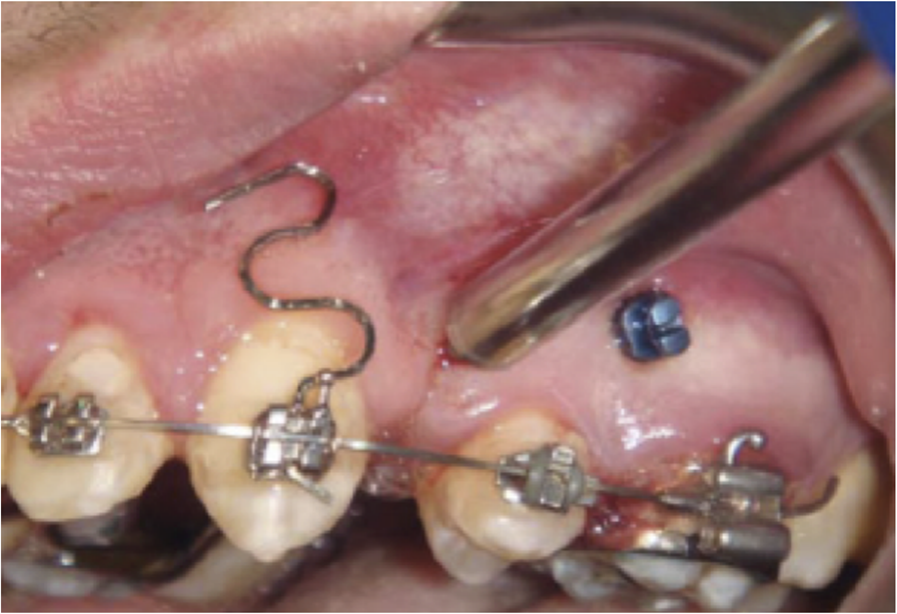
Propel handheld disposable device used for MOPs (Alikhani, 2013)

Alikhani [25] used MOPs (Propel) in a clinical trial in 20 adults with Class II division 1 malocclusions. Three flapless microperforations were performed under local anaesthesia distal to the canines, 6 months after the premolar extractions, in order to accelerate tooth movement (Fig. 7). The drawbacks of the technique are:

1. The extent of the injury created to the bone is minimal and therefore the RAP effect may not be as long as it is intended.
2. It is a blind technique and pre-planning of the position of the MOPs is crucial to avoid injury to the roots.
3. The inability to graft hard or soft tissues during the procedure to correct and reinforce the periodontium.

4- It is time consuming especially in the mandible because of the thick cortex and needs to be repeated frequently adding cost and chair time to treatment.

## Review of the Literature

There have been case reports, animal and clinical studies published on all the above techniques, especially in the past 10 years, due to the increased popularity of surgically accelerated orthodontics (Table 1).

**Table 1:** Publications on surgically accelerated orthodontics in chronological order

Author(s) and year of publication	Type of subjects	Number of subjects	Control group and type	Procedure being assessed	Type of publication
**Liou and Huang, 1998** **[**14**]**	Human	15	No	Rapid canine retraction/dental distraction	Clinical study
**Wilcko and Wilcko, 2001** **[**18**]**	Human	2	None	PAOO	Case report—2 cases
**Kisnisci et al., 2002** **[**36**]**	Human	11	No	Rapid canine retraction/dental distraction	Clinical study
**Bilodeau, 2003** **[**35**]**	Human	1	No	Rapid canine retraction/dental distraction	Case report
**Iseri et al., 2005** **[**37**]**	Human	10	No	Rapid canine retraction/dental distraction	Clinical study
**Lee et al., 2007** **[**30**]**	Human	65	Yes—conventional orthodontics	Corticotomy, segmental osteotomy	Clinical study
**Iino et al., 2007** **[**32**]**	Beagle dogs	12	Split mouth design	Corticotomy	Animal study
**Nowzari et al., 2008** **[**27**]**	Human	1	None	PAOO	Case report
**Wilcko and Wilcko, 2009** **[**28**]**	Human	2	None	PAOO	Case report—2 cases
**Wilcko and Wilcko, 2009** **[**29**]**	Human	3	None	PAOO	Case report—3 cases
**KIm et al., 2009** **[**19**]**	Cat	16	Yes	Corticision	Animal study
**Dibart et al., 2009** **[**22**]**	Human	1	None	Piezocision	Case report
**Mostafa et al., 2009** **[**33**]**	Beagle dogs	6	Split mouth design	Corticotomy	Animal study
**Sanjideh et al., 2010** **[**34**]**	Foxhound dogs	5	Split mouth design	Corticotomy	Animal study
**Teixeira et al., 2010** **[**55**]**	Sprague-Dawley rats	48	Yes	Soft tissue flap and osteoperforations	Animal study
**About-Ela et al., 2011**	Human	13	Split mouth design	Corticotomy	Clinical study
**Keser and Dibart, 2011** **[**40**]**	Human	1	None	Piezocision	Case report
**Keser and Dibart, 2013** **[**41**]**	Human	1	None	Piezocision	Case report
**Alikhani et al., 2013** **[**25**]**	Human	20	Yes—conventional orthodontics	Microosteoperforations	Clinical study
**Murphy et al., 2014** **[**38**]**	Wistar Rats	44	Yes	Corticision	Animal study
**Milano et al., 2014** **[**42**]**	Human	1	None	Piezocision	Case report
**Dibart et al., 2014** **[**53**]**	Sprague-Dawley rats	94	Yes	Piezocision	Animal study
**Murphy et al., 2016** **[**39**]**	Wistar rats	44	Yes	Corticision	Animal study
**Aksakalli et al., 2016** **[**44**]**	Human	10	Split mouth design	Piezocision	Clinical study
**Charavet et al., 2016** **[**45**]**	Human	24	Yes—conventional orthodontics	Piezocision	Clinical study
**Abbas et al., 2016** **[**46**]**	Human	20	Split mouth design	Two groups for canine retraction: corticotomy and piezocision	Clinical study
**Dibart et al., 2016** **[**68**]**	Ex-vivo calvarial bone organ culture—mice	276	Yes	Groups: piezoelectric knife, bur, handheld screw device	Animal study
**Uribe et al., 2017** **[**67**]**	Human	29	Yes—conventional orthodontics	Piezotome-corticisions	Clinical study
**Sugimori et al., 2018** **[**56**]**	Wistar Rats	50	yes—orthodontic force only	Microosteoperforations	Animal study
**Alkebsi et al., 2018** **[**58**]**	Human	32	Yes—conventional orthodontics	Microosteoperforations	Clinical study
**Chan et al., 2018** **[**61**]**	Human	20	Yes—conventional orthodontics	Microosteoperforations	Clinical study
**Attri et al., 2018** **[**59**]**	Human	60	Yes—conventional orthodontics	Microosteoperforations	Clinical study
**Hou et al., 2019** **[**43**]**	Human	1	None	Piezocision	Case report
**Strippoli et al., 2019**	Human	24	Yes—conventional orthodontics	Piezocision	Clinical study
**Van Gemert et al., 2019** **[**57**]**	Beagle dogs	13	Split mouth design	Microosteoperforations	Animal study
**Sivarajan et al., 2019** **[**60**]**	Human	30	Split mouth design	Microosteoperforations	Clinical study
**Charavet et al., 2019** **[**54**]**	(Oncins France Strain A) Rats	60	Yes—orthodontic force only	Piezocision	Animal study
**Hatrom et al., 2020**	Human	26	Yes—conventional orthodontics	Piezocision	Clinical study
**Khlef et al., 2020** **[**52**]**	Human	40	No	Two groups: corticotomy done by using piezoelectric knife with flap and piezocision( defined as flawless corticotomy)	Clinical study
**Hannequin et al., 2020** **[**71**]**	Human	1	None	Corticotomy done by using piezoelectric knife with flap	Case report
**Hatrom et al., 2021** **[**50**]**	Human	23	Yes—conventional orthodontics	Piezocision	Clinical study
**Alvarez et al., 2021** **[**51**]**	Human	36	Yes—conventional orthodontics	Piezocision	Clinical study
**Kernitsky et al., 2021** **[**69**]**	Sprague-Dawley rats	18	Yes	Groups: deep and shallow piezoelectric decortications	Animal study
**Sharon et al., 2021**	Human	30	Yes—conventional orthodontics	Microosteoperforations	Clinical study

### Corticotomy/PAOO

#### Case reports

Wilcko and Wilcko [18] described the PAOO technique with a report of two cases in 2001. A Class I case with severe crowding who was 24 years old and a Class I case with moderate to severe crowding who was 17 years old were treated using PAOO. Orthodontic adjustments were made at two-week intervals, approximately after the surgery and the treatments were completed within 6 months and 2 weeks, which reduced treatment time by 75%. The first patient also required bone grafting, with re-entry undertaken 15 months following surgery (8.5 months following debond). Clinical observation demonstrated acceptable maintenance of the alveolar crest height and an increased buccal bone thickness. Despite the canine and premolars in this area being expanded buccally by more than 3 mm, there had been an increase in the buccolingual thickness of the overlying buccal bone. The authors suggested that the rapid expansive tooth movements with no significant apical root resorption may be attributed to the osteoclastic or catabolic phase of the RAP. The conclusion was that corticotomies resulted in transient osteoporosis, accelerating tooth movement. The potential for grafting was an important addition to corticotomy-facilitated orthodontics, especially in cases with preexisting defects.

Nowzari et al. [27] used the PAOO technique in a Class II division 2 case, aged 41 years, in order to accelerate orthodontic movement. Treatment was completed in 8 months, with re-entry after 4 months. The maxillary and mandibular alveolar ridges maintained the original thickness and configuration, despite proclination of the incisors. They concluded that PAOO was an effective treatment approach in adults, reducing both treatment time the risk of root resorption.

Wilcko and Wilcko [28] presented two more cases treated with PAOO, including the stability of the results up to 8 years of retention, in 2009. The first patient had a Class I malocclusion with crowding of both arches and severe maxillary arch constriction. This case was completed in just over 6 months, with 8 mm of transverse arch expansion. The second patient had moderate anterior crowding. Her treatment time was 7 months. Both patients showed thickening of the alveolar housing at re-entry, and all the dehiscences and fenestrations were filled with bone. The authors stated that, “The accelerated osteogenic orthodontics technique provides for efficient and stable orthodontic tooth movement. Frequently, the teeth can be moved further in one third to one fourth the time required for traditional orthodontics alone”. In the same year, Wilcko and Wilcko [29] published three more cases that required first premolar extractions and canine retraction. The first case had a Class I molar relationship; maxillary first bicuspids and mandibular canines extracted, and the PAOO surgery followed. Her treatment time was 9 months. The other patient had bimaxillary protrusion with an 8-mm anterior open bite and moderate labial segment crowding. The first premolars were removed, the canines were retracted, and treatment was completed in 9 months. Another case presented with a Class II malocclusion. Following PAOO surgery the treatment duration was 12 months. The authors’ conclusion was that the PAOO technique allowed teeth to be moved 2-3 times faster, 1/4 to 1/3 of the time required for orthodontic treatment, and side effects of orthodontic movement such as root resorption and relapse can be reduced with PAOO.

#### Human studies

Lee et al. [30] compared the treatment outcomes of orthodontic treatment only, anterior segmental osteotomy or corticotomy-assisted orthodontic treatment for the management of bimaxillary dentoalveolar protrusion. From the sample of 65 patients, 29 were treated with conventional orthodontics and anchorage reinforcement using a transpalatal arch with or without headgear. Twenty were treated with corticotomy-assisted orthodontic treatment with skeletal anchorage in the maxilla and anterior segmental osteotomy in the mandible; and 16 with anterior segmental osteotomy in the maxilla and mandible. The mean treatment duration for the conventional orthodontic group was the longest. The corticotomy-assisted orthodontic treatment can be advantageous for adult patients concerned with prolonged treatment duration. Anterior segmental osteotomy is recommended for bimaxillary dentoalveolar protrusion patients with increased gingival exposure on smiling, skeletal prognathism, relatively normal incisor inclination, and relative deficiency in chin prominence.

Aboul-Ela et al. [31] compared miniscrew implant-supported maxillary canine retraction with and without corticotomy-facilitated orthodontics in 13 adult patients with Class II division 1 malocclusions. A split-mouth investigation design was used. Canine retraction rate was two times faster on the test sides in the initial 2 months. It was 1.6 times faster in the third month and almost the same (1.06 times higher) by the end of month 4. No statistically significant difference was found between preoperative and postoperative measurements in relation to probing depth, attachment loss, gingival recession, or plaque index.

#### Animal studies

Iino et al. [32] did a split-mouth study on twelve beagle dogs to measure tooth movement rate and the post-corticotomy bone reaction. Four second bicuspids were removed. A 16-week waiting period was given for complete callus formation and mineralization prior to the corticotomy. It was found that movement was constant and faster, and the total amount of tooth movement on the experimental side was double. Root resorption was found after 4 and 8 weeks on the control side, but no root resorption was observed on the experimental side. Following the corticotomies, orthodontic tooth movement increased for at least 2 weeks. The authors suggested that this may have been brought about by rapid alveolar bone reaction in the bone marrow cavities, which led to less hyalinization of the periodontal ligament on the alveolar wall.

Mostafa et al. [33] investigated corticotomy-facilitated orthodontic tooth movement using miniscrews on an animal model. The maxillary second premolars of six beagle dogs were removed. Miniscrews were inserted between the roots of the third premolars and the first molars bilaterally. Corticotomy was performed on the right side. Notches were placed on the two teeth allowing the distance between the first and third premolars to be measured weekly, and histological samples were acquired. They found that tooth movement was twice as fast on the experimental side. Histology demonstrated greater activity and more extensive remodelling of the bone in the corticotomy group. This suggested that accelerated tooth movement following corticotomy may be due to increased bone turnover, based on the RAP.

Sanjideh et al. [34] assessed the effect of corticotomy on a dog model in a split-mouth study design. The authors investigated whether corticotomy procedures increase tooth movement and the effects of a second corticotomy procedure after 4 weeks on the rate of tooth movement. Second premolars were extracted in the upper arch and third premolars in the lower arch. One upper and one lower quadrant had corticotomy only on the extraction day. Another upper quadrant had a second procedure carried out 28 days later. The final quadrant was the control. Tooth movement on the test side was twice that of the control side after 56 days. The authors concluded that corticotomy significantly increased orthodontic tooth movement. Performing a second corticotomy procedure after four weeks maintained higher rates of tooth movement over a longer duration. It also produced greater overall tooth movement than performing just one initial corticotomy, although the difference was small. Therefore, the second corticotomy procedure was not deemed to be justified.

### Rapid canine retraction/dental distraction

#### Case report

Bilodeau published a case report in 2003 [35] demonstrating the application of dental distraction on a 37-year-old male. The dental distraction was used only on the upper left side; the patient was seen every three days during retraction. The canine retraction was completed in 25 days. He reported that the patient experienced no discomfort with the device, and the tooth tested vital to ice at the end of the distraction.

#### Human studies

Liou and Huang [14] had 15 orthodontic patients (26 canines, including 15 maxillary and 11 mandibular) requiring first premolar extraction and canine retraction. Distal to the canine, the interseptal bone was undermined with a bone bur, with grooves vertically inside the extraction socket, along the buccal and lingual sides and extending obliquely towards the socket base (Fig. 1). An intraoral distraction device was placed to distract the canine distally into the extraction space. This was activated 0.5-1.0 mm/day immediately after the extraction. The upper and lower canines were distracted bodily 6.5 mm into the extraction space within 3 weeks. New alveolar bone was generated and remodelled rapidly in the mesial periodontal ligament of the canine during and after the distraction. Radiographic examination revealed that apical or lateral surface root resorption of the canine was minimal. No periodontal defect or endodontic lesion was observed throughout or after distraction. They concluded that the PDL could be rapidly distracted without complications.

Kisnisci et al. [36] modified the rapid canine distraction by adding horizontal and vertical osteotomies surrounding the canines, in order to achieve rapid movement in the dentoalveolar segment, in compliance with the principles of distraction osteogenesis. They used dentoalveolar distraction on 11 patients. The first premolar was extracted, and the buccal bone was carefully removed. After mucosal incisions, cortical holes were made in the alveolar bone from the canine to the second premolar, curving apically to pass 3-5 mm from the apex. The holes were connected round the root. Fine osteotomes were advanced coronally. Following the first premolar extraction, the buccal bone was removed between the outlined bone cut at the distal canine region anteriorly and the second premolar posteriorly. Larger osteotomes were used to fully mobilize the alveolar segment with the canine. The palatal shelf was preserved, but the apical bone near the sinus wall was removed, leaving the sinus membrane intact to avoid interferences during the active distraction process. Osteotomes along the anterior aspect of the canine were used to split the surrounding bone around its root from the palatal or lingual cortex and neighbouring teeth. The transport dentoalveolar segment with the canine tooth also included the buccal cortex and the underlying medullary bone that envelopes the canine root, leaving an intact lingual or palatal cortical plate and the bone around the apex of the canine. Distraction was started the same day at the rate of 0.4 mm twice a day and continued until adequate movement of the canine teeth was achieved. The authors reported good patient tolerance with the distraction rate and the device. No root resorption, discoloration, dental ankylosis, loss of vitality, or anchorage loss in the second premolar and first molar teeth was detected.

Iseri et al. [37] used ten subjects to study the dentoalveolar distraction of twenty maxillary canines. First premolars were extracted, and the dentoalveolar distraction surgical procedure performed as described by Kisnisci, and a custom-made intraoral, rigid, tooth-borne distraction device was placed. The canines were moved rapidly into the extraction sites in 8–14 days (rate of 0.8 mm/day). There was no clinical or radiographic evidence of complications observed in any of the patients.

### Corticision

#### Animal studies

Kim et al. [19] evaluated the biologic effects of corticision on alveolar remodelling in orthodontic tooth movement using cats. Group A (control) had orthodontic force only. Group B had corticision and orthodontic forces. Group C had corticision together with orthodontic forces and periodic mobilization. The cats in the control group received no surgery. Each group was also divided into 4 subgroups, according to the force application duration (7,14 ,21 and 28 days). The canines were retracted with a 100*g* force, and the first and second molars were used for anchorage. The test groups (B and C) had corticision on the mesial and distal buccal, and distal-palatal aspects of the maxillary canines, using a reinforced surgical blade and mallet for bone decortication. In group C, mobilization was carried out immediately following corticision. This was repeated every 3 days. The authors observed extensive direct resorption of the bundle bone with rapid removal of hyalinized tissue in group B, compared to the controls. The mean apposition area of new bone was 3.5 times higher in group B than in the control group at 28 days. There was no obvious root resorption or any other pathological change in the corticision groups. There were no differences between groups B and C, suggesting periodic mobilization was not sufficient to increase RAP duration. The authors explained that RAP can reduce the lag phase of tooth movement by stimulating hyalinized tissue removal, thereby resulting in reduced orthodontic treatment time.

Murphy et al. [38] used two different force magnitudes on the rate of tooth movement on rats. They assessed the effects of corticision on alveolar bone remodelling 14 days following the start of orthodontic movement. Forty-four rats were divided into four groups, receiving either a heavy 100*g* or a light 10*g* force, with or without corticision. The corticision procedure was performed at appliance placement with force applied from the upper first molars to the central incisors. A week later spring reactivation was undertaken and corticision repeated in the corticision groups. The contralateral side was used as a control in every groups. Microcomputed tomographic analysis was carried out on day 14 to assess quantitatively for any bony changes and to evaluate the tooth movement. Histomorphometric analysis was performed to evaluate osteoclastic activity and whether it related to the changes in bone density. No significant differences in the microcomputed tomography data were found. The authors concluded that, regardless of the force magnitude, a flapless surgical insult in the mesiopalatal aspect of the first molar with a single-site corticision was unable to induce clinical or histologic changes after 2 weeks of orthodontic tooth movement. Histological analysis showed a significantly reduced bone volume fraction for the group with light loads compared with the other groups.

Murphy et al. [39] repeated the experiment in 2016 to assess the effect of corticision with different force magnitudes on root resorption. Two-dimensional (histomorphometric) and three-dimensional (volumetric, micro-focus X-ray computed tomography [micro CT]) analysis of root craters were performed on maxillary first molars. The results suggested that corticision had a small and statistically insignificant effect in reducing root resorption. No difference in root resorption was observed between 10 and 100 g of force measured by two-dimensional histomorphometry or three-dimensional micro CT.

### Piezocision

#### Case reports

**Fig. 8:**
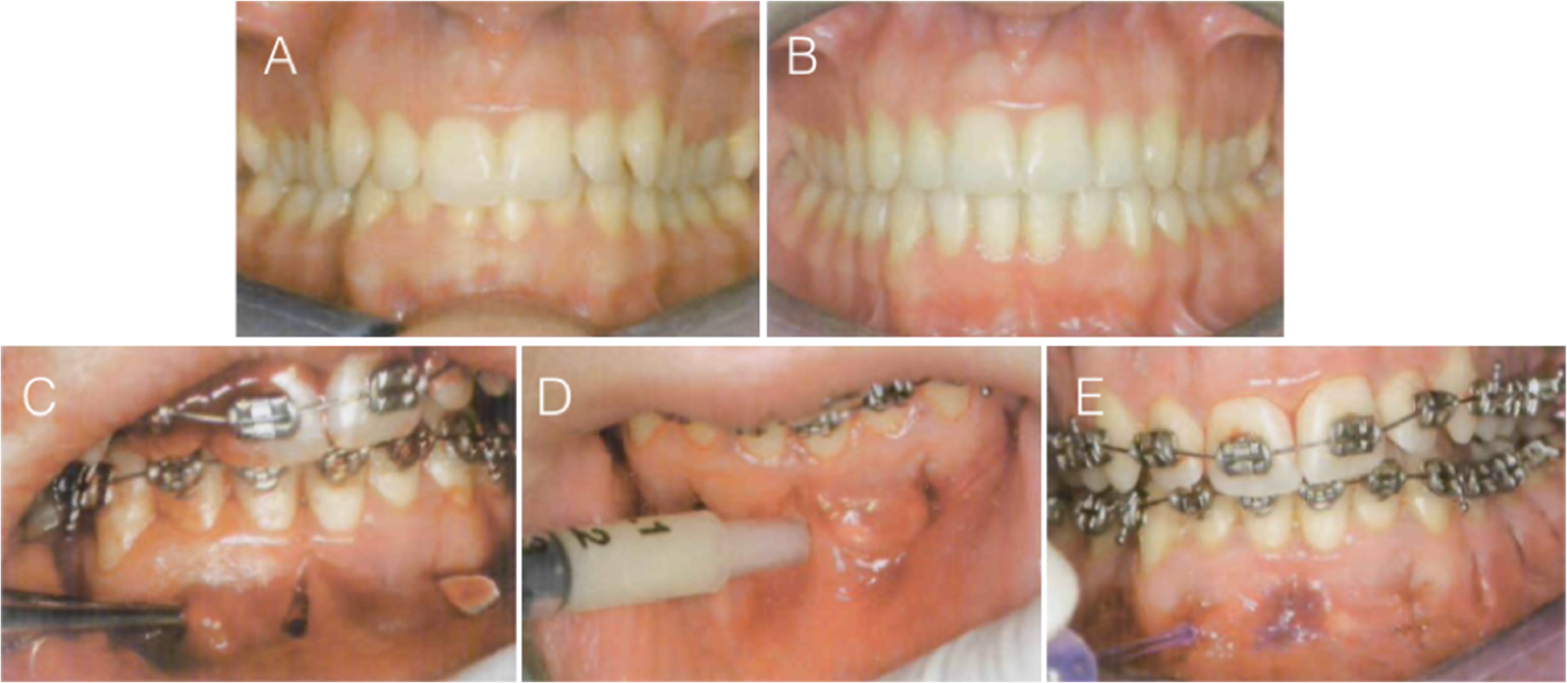
Case report (Dibart, 2009). **a** Pretreatment frontal view. **b** Post-treatment frontal view. **c** Tunnelling of areas to be grafted with bone. **d** Bone grafting. **e** Sutures in place

Piezocision™ was first described by Dibart in 2009, in a case report [22]. The patient had a Class II division 2 incisor relationship with slight maxillary retrusion. Piezocision™ was undertaken one week after the orthodontic appliances were placed. Ten vertical interproximal microincisions were made in both jaws. Corticotomies of approximately 3-mm depth were made with a piezoelectric knife. The areas requiring expansion were tunnelled and bone grafts were placed. The treatment was completed in 17 weeks. The author concluded that piezocision is a minimally invasive alternative to the PAOO, with the total surgical time less than an hour. It was suggested that the approach led to short orthodontic treatment time, minimal discomfort, and good patient acceptance, as well as stronger periodontium, because of the added grafting (Fig. 8).

Keser and Dibart [40] published a case report demonstrating a case treated with an Invisalign appliance using piezocision to accelerate the treatment. The case report showed how to tackle the two major issues when it comes to adult orthodontics - the use of a discreet appliance in a timely manner. The authors illustrated how, in selected cases, piezocision combined with Invisalign treatment can be used to treat adults successfully. Another case report by Keser and Dibart [41] in 2013, used sequential piezocision for Class III correction, significantly reducing the treatment duration and the utilization of the RAP in a sequential manner helped with the complex tooth movement and anchorage requirements. The patient was 25 years of age, female, with a Class III malocclusion, narrow maxilla and crossbites of multiple teeth. She had 7.5 mm of upper crowding and 2 mm crowding in the lower arch. The option to have extractions in the upper arch and orthognathic surgery as the ideal treatment plan was provided, but the patient decided against it. Treatment was started by bracketing the maxillary arch and the piezocision was performed. Only after 10 weeks, once the upper alignment was achieved, the orthodontic appliances were placed in the mandible and the lower jaw piezocision was undertaken. The orthodontic treatment was completed in 8 months. Fixed and removable retainers were used. Piezocision was used successfully in a sequential manner to correct the Class III malocclusion.

A case report was published by Milano et al. [42] to demonstrate the use of a surgical guide, to reduce the risk with piezocision where there is close root proximity. The authors demonstrated that the depth and location of corticotomies can be precisely planned using a three-dimensional model of the arch. A surgical guide can be fabricated and used to prevent damage to the dental roots. Another case was presented by Hou et al. [43] where a 3D computer-assisted piezocision guide was used to minimize the risk of surgical complications. The guide was designed as translucent for increased visibility, rigid for enhanced support and porous for profuse intraoperative irrigation.

**Fig. 9:**
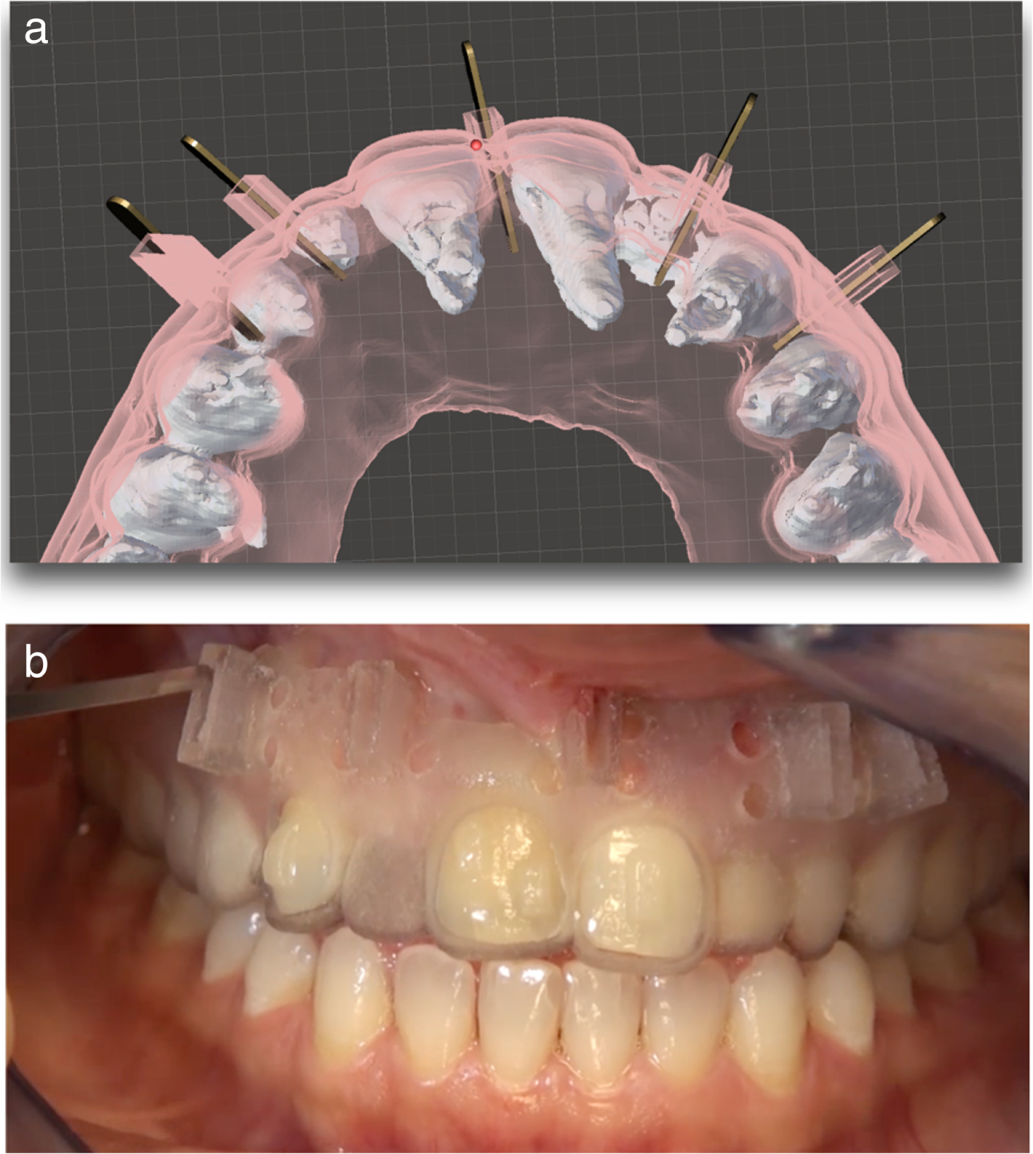
**a** Piezocision surgical guide planning, showing the ideal angulation/insertion of the piezoelectric knife (design by Dr. Keser). **b** Application of the surgical guide digitally created to guide piezocision surgery for a patient being treated by clear aligners

Figure 9 a shows another design of a piezocision guide that was used for a patient who was being treated with Invisalign (not published). Software was used to superimpose the CBCT images of the patient and the guide design, to determine the position of the piezocision cuts and the angulation of the piezoelectric knife in order to avoid any damage to the roots. The height of the cutting holes was planned to be tall enough to prevent any wobble of the piezoelectric knife during the procedure. Irrigation holes were present for water cooling. Using a guide like this shortened the piezocision procedure time and also made the positioning of the cuts extremely accurate and avoided any injuries to the roots (Fig 9b).

#### Human studies

Aksakalli et al. [44] investigated the effectiveness of piezocision in canine distalization in Class II patients. They recorded the transverse changes, the post-distalization gingival status, and mobility scores in a split-mouth design, where first maxillary premolars were extracted and canines were distalized. The initial stage was alignment of the teeth. After the alignment was complete, piezocision was performed on one side. Two buccal vertical incisions were made, each approximately 10 mm, to create decortications at a depth of 3 mm. Elastometric chains with a force of 150*g* were used for distalization, adjustments were done every 2 weeks, and elastomeric chains were replaced at each appointment until Class I canine relationship was achieved. Measurements of movement were compared between the experimental and control group before and after distalization, as were gingival scores and also mobility. Three-dimensional analysis of the study casts showed significant differences in tooth movement, less anchorage loss and greater canine distalization in the experimental group. Also, the distalization time was shortened in the experimental group. There was no difference between the two groups for transversal changes, mobility or gingival indices. They concluded that piezocision reduced canine distalization time by half, and decreased the anchorage loss for posterior teeth, and piezocision does not have any adverse effects on periodontal health.

Charavet et al. [45] compared conventional orthodontic treatment to piezocision-assisted orthodontic treatment in an adult group of patients with crowding. Piezocisions were performed 1 week after the orthodontic appliances were placed, and follow up appointments were made every 2 weeks. The authors compared the treatment duration, periodontal health, alveolar crest changes, analgesic intake in both groups. The treatment time significantly reduced by 43%in the piezocision group. Radiographic and periodontal parameters were similar following treatment in both groups, no increase in root resorption was observed in either group. Scars were observed in 50% of the patients in the piezocision group. The piezocision group demonstrated higher patient satisfaction and smaller variations in the duration of treatment. The authors concluded that piezocision is an effective method in accelerating orthodontic tooth movement. The risk of residual scarring should be kept in mind, especially in patients with a high smile line. Abbas et al. [46] compared the efficiency of corticotomy facilitated orthodontics and piezocision in rapid canine retraction. They used a piezotome in both groups to create the decortication. In the corticotomy side they removed the bundle of bone from the medial wall of the extraction socket of the premolar to decrease the resistance to tooth movement, in a similar fashion described in rapid canine distraction by Liou in 1998 [14]. They concluded that both corticotomy-facilitated orthodontics and piezocision are effective treatment alternatives that reduce canine retraction duration and root resorption in adults. Corticotomy-facilitated orthodontics is 1.5-2 times faster than conventional orthodontics. Piezocision was 1.5 times faster than conventional orthodontics.

Strippoli et al. [47] carried out a clinical trial to compare the duration of orthodontic treatment with peizocision versus conventional orthodontic treatment. They also evaluated the safety of the piezocision and assessed the inflammatory process, periodontal health and soft tissue healing during the first 6 months in the piezocision group. They found that the duration of treatment was significantly decreased by 54%in the piezocision group compared with the conventional orthodontics group. Most patients indicated that they would be willing to undergo a piezocision procedure again. Minimal effects on periodontal parameters were observed despite the presence of some gingival scars. Although not statistically significant, pro inflammatory markers showed clinical peaks at 3–5 and 16 weeks following surgery. These were in agreement with a systematic review by Yi et al. [48] and confirmed that the piezocision-assisted orthodontic tooth movement can be considered to be a safe procedure. Hatrom et al. [49] used a randomized controlled clinical trial to compare the en masse retraction with or without piezocision, to assess the type of tooth movement. They also evaluated root integrity after the retraction and recorded the pain levels. Piezocision cuts were carried out on the buccal aspect of the jaw and in addition a piezotome was used to remove the bone from the extraction socket distal to the canine root and palatal side of the socket. They concluded that piezocision enhanced the amount of en masse retraction by two times, with less root resorption; the piezocision group showed a reduced amount of incisor tipping and root resorption. Postoperative pain experienced by patients was moderate. The same group of investigators published another study where they evaluated the pulp volume changes after piezocision-assisted tooth movement [50]. They used two groups of patients requiring orthodontic treatment with bilateral maxillary first premolar extractions and en masse retraction: 1- extraction with piezocision, 2- only extraction. At the end of the en masse retraction phase, pulp volume was significantly reduced in all six anterior teeth in both groups. The decrease in pulp volume was not statistically different between the groups. They also concluded that the degree of change in pulp volume did not appear to be related to the amount of root resorption. Alvarez et al. [51] compared the extent of buccal bone defects and transverse tooth movement of mandibular lateral segments in patients after orthodontic treatment with and without piezocision. The study sample consisted of 36 patients with moderate mandibular anterior crowding. No significant differences were found in buccal dehiscences and transverse tooth movement of the mandibular lateral segments. In both groups dehiscences, buccal inclination and arch width increased significantly at the end of the treatment. The results were similar to those of Aksakalli et al. [37], who did not find any intercanine maxillary transverse differences between the piezocision group and control group when retracting canines after premolar extraction.

In a recent randomized controlled clinical trial, Khlef at al [52]. evaluated the efficacy of corticotomies, with and without flaps, which were performed using a piezoelectric knife. They had forty Class II division 1 patients requiring maxillary first premolar extractions. One group was treated by flap elevation and using a piezoelectric knife for decortications, and the other group was treated without a flap and piezoelectric corticotomies in en masse retraction by using miniscrews for anchorage. No significant differences between the groups were found in terms of skeletal, dental, and soft tissue variables, as well as the amount of external apical root resorption. They concluded that corticotomy-assisted en masse retraction, with or without flaps, led to improvements in skeletal structures, facial profile and resulted in sufficient retraction of maxillary anterior teeth, and that neither technique caused significant root resorption.

#### Animal studies

**Fig. 10:**
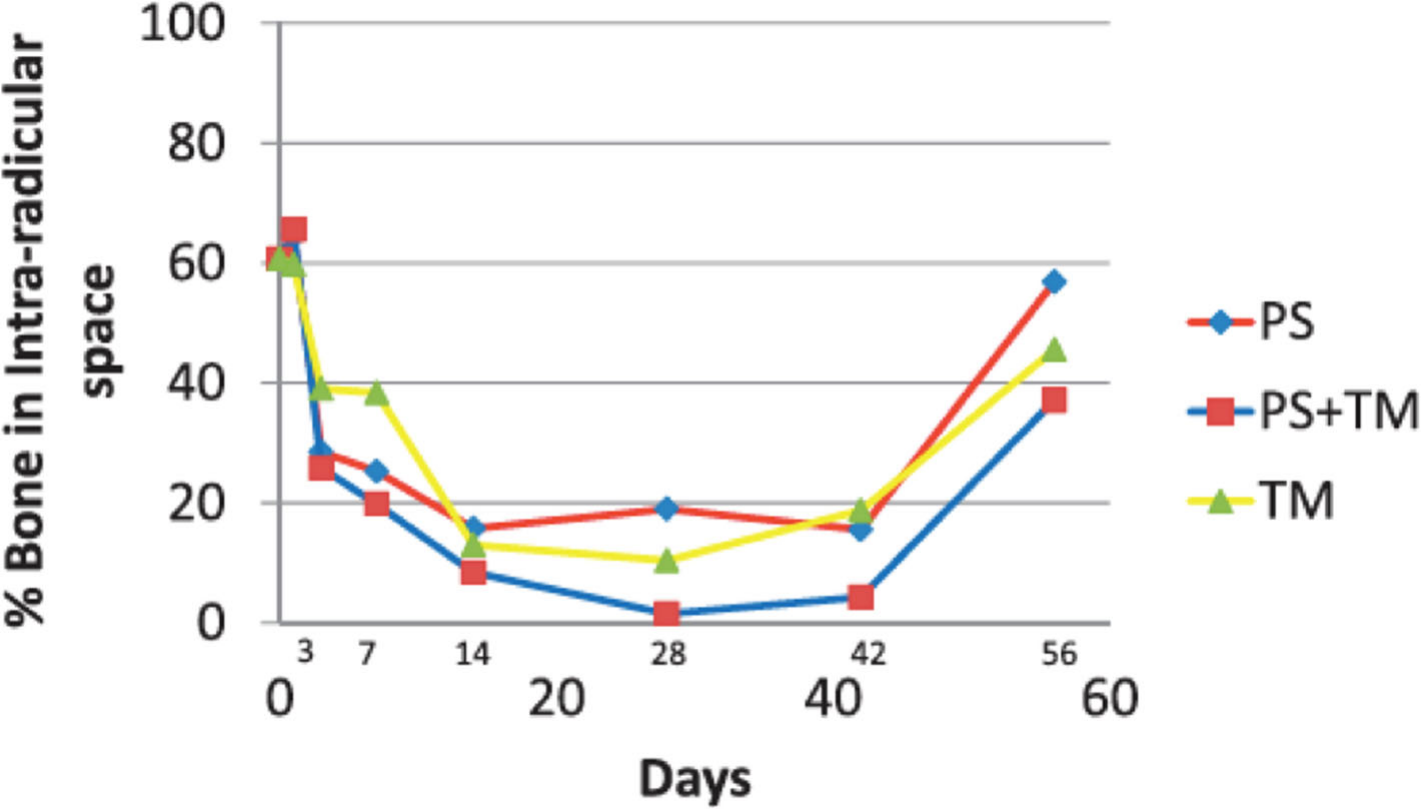
Percentage of bone demineralization over time in the three experimental groups: piezocision alone (PS), piezocision and tooth movement (PS+TM), tooth movement alone (TM) (from Dibart et al. [54].)

Dibart et al. [53] used 94 rats to study the effects of piezocision on bone, with and without tooth movement. The four groups were no active treatment, tooth movement only, piezocision only, and tooth movement with piezocision. Alveolar bone histomorphometry after piezocision was assessed, as were catabolic activity and extent of demineralization at various stages from 1 to 56 days. An increase in the rate of bone resorption following piezocision began on day 3 and continued to day 14, after which the movement of the teeth became the driving force for bone resorption. The increase in osteoclast numbers were significantly greater in the piezocision and tooth movement groups. The greatest level of osteoclastic activity in the tooth movement only group was on the third day, which decreased afterwards. The results showed that piezocision increases the rate of tooth movement and suggested that the increased osteoclastic activity following decortication is extended by the synergistic relationship between piezocision and orthodontic tooth movement (Fig. 10). Charavet et al. [54] also used rats to study the alveolar bone tissue response following piezocision to demonstrate the underlying biological mechanism. Sixty rats were randomly distributed between a control group (conventional tooth movement) and an experimental group (piezocision-assisted tooth movement). Tissue, cellular, and molecular analyses were performed at 7, 28, and 42 days. The results showed that orthodontic tooth movement was 1.8 times faster in piezocision group at day 42. The bone volume fraction was significantly decreased in the piezocision group on day 7 and 28, but there was no difference on day 42. On day 7, the number of osteoclasts were significantly higher in the piezocision group. There was no difference between the two groups in the number of osteoclasts involved in root resorption. This study demonstrated the effectiveness of piezocision to accelerate tooth movement.

### Micro-osteoperforations

#### Animal studies

Teixeira et al. [55] used rats to study the effects of osteoperforations using burs. The authors hypothesis was that stimulation of the expression of inflammatory cytokines, via osteoperforations of cortical bone, increases the rate of bone remodelling and tooth movement. The investigation used 48 Sprague-Dawley rats with four groups: control, orthodontic force applied by a coil spring (O), orthodontic force with a soft tissue flap (OF, the flap was raised around the left first molar), or the same as the OF group but with shallow perforation of the cortical plate (OFP). This involved raising a soft tissue flap around the left first molar and three shallow perforations, with a round bur, of 0.25 mm diameter and depth, placed 5 mm mesial to the left first molar. The results showed that osteoperforations increase the rate of tooth movement, by increasing the rate of bone remodelling and general osteoporosity. The O and OF groups had a statistically lower expression of 21 cytokine receptors than the OFP group. Another animal study from Japan that was undertaken by Sugimori [56] on rats focused on the inflammation, cell proliferation and apoptosis of periodontal ligament cells. The experiment period was 14 days and demonstrated that MOPs accelerated orthodontic tooth movement with a decreased bone mineral density and bone volume/tissue volume ratio and an increased expression of THF-alpha and proliferation and apoptosis of compressed PDL cells. The authors concluded that MOPs may be effective in accelerating orthodontic tooth movement and reducing the traditional orthodontic treatment time. Van Gemert et al. [57] used a split-mouth design on beagle dogs. They wanted to determine how far the effects of MOPs can extend within the bone. They would quantify the damage caused and the short-term bony adaptations that occur in and around the injury site. They used 13 beagle dogs and evaluations were performed 2 and 4 weeks after MOPs. They concluded that the demineralization effects of MOPs on bone are transient and are not evident after 4 weeks. The principal effects do not extend more than 1.5 mm from the MOP and normal tabular healing occurs following MOP placement.

#### Human studies

Alikhani [25] in 2013 investigated the effect of micro-osteoperforations (MOPs) on the rate of tooth movement and on the expression of inflammatory markers. Twenty adults with Class II division 1 malocclusions were included in the study and divided into control and experimental groups. The experimental group received MOPs on one side of the maxilla, while the control group only had orthodontic treatment. All subjects had the maxillary first bicuspids removed. MOPs were done after the levelling and alignment were completed and the cases were ready for retraction. Three MOPs were performed in the experimental group under local anaesthesia, distal to the canines, prior to the retraction undertaken with a disposable device (by PROPEL Orthodontics, NY). Each perforation was 1.5 mm in width and 2-3 mm in depth. Orthodontic force was applied with a coil spring connected from a TAD to a power arm on the cuspid bracket. The study was concluded after 28 days of canine retraction. Discomfort levels were provided by the participants at different time points. There was no difference between groups at any time point. After 24 h of canine retraction, the levels of chemokynes increased significantly in both groups. In the control group, by day 28, only IL-1 was still significantly higher than before retraction. In the test group IL-1α and IL-1β levels were higher compared to before retraction. The study model measurements at day 28 showed that canine retraction in the experimental group to be 2.3 times higher compared to the control group. This was accompanied by a significant increase in the inflammatory markers. They concluded that MOPs are an effective and comfortable technique for the acceleration of tooth movement.

Alkebsi et al. [58] used a split-mouth design to investigate the effect of MOPs on the rate of tooth movement. This randomized controlled trial used 32 patients who needed maxillary first premolar extractions. Miniscrews were used to support anchorage and for canine retraction. Three MOPs were performed using miniscrews on the buccal bone distal to the canines on the randomly selected side. They found no statistically significant difference in the rates of tooth movement between the MOP and the control sides at any time point. They concluded that three MOPs were not effective in accelerating tooth movement at any time point. Attri et al. [59] had 60 subjects requiring en-masse retraction following first premolar extractions. The experimental group received MOP distal to the canines, and they were compared with a control group treated with identical brackets without MOP. They concluded that MOP appears to increase tooth movement rate, but with no difference in the perception of pain. Monthly space closure rate was 0.73 to 0.89 mm in the MOP group and 0.49 to 0.63 mm in the control group. Sivarajan et al. [60] used 30 subjects to assess the effect of MOPs in three different canine retraction groups. Group 1: MOP 4-weekly upper arch/8-weekly lower arch, Group 2: MOP 8-weekly upper arch/12-weekly lower arch, and Group 3: MOP 12-weekly upper arch/4-weekly lower arch, with measurements undertaken at 4-week intervals over 16 weeks. Mean overall canine retraction was 4.16 mm with MOP and 3.06 mm without. All the MOP groups exhibited significantly higher canine distalization than the control group. The subjects reported pain associated with MOP, with 60% classifying it as moderate and 15% as severe. The main impact of this reported pain was related to chewing and speech. The authors concluded that MOP can increase overall mini-implant supported canine retraction over a 16-week period of observation, but that this difference is unlikely to be clinically significant. Chan et al. [61] looked into the effects of MOPs on orthodontic root resorption. In this prospective controlled trial, the authors used 20 subjects requiring extraction of the upper first premolars as part of their orthodontic treatment. A 150*g* force was applied to both premolars, and 5-mm depth MOPs were applied mesially and distally in the mid-root region of the experimental premolar. The premolars were extracted after 28 days and microcomputer tomography was used to measure the volumes of the root resorption craters. The results showed greater orthodontic root resorption where MOPs were applied. Some of the points mentioned in this study were related to the RAP effect and that enhanced alveolar bone turnover associated with an increased osteoclastic activity was found to exacerbate the root resorption process [62, 63]. Also, it is possible that excessive localized activation of osteoclasts, as seen after surgical procedures such as MOPs [55, 64], can lead to an increase in odontoclastic activity [62]. The authors concluded that further research should be undertaken on patients undergoing a full course of orthodontic treatment and that repair of root resorption craters after MOPs should be investigated.

Most recently, Shahrin et al. [65] investigated the effectiveness of MOPs in overall time taken for alignment of maxillary anterior crowding and evaluated the alignment improved percentage within 6 months between MOPs and cantor groups. They used 30 subjects with moderate upper labial segment crowding. All participants had their first premolars extracted. The intervention group received 3-mm-deep MOPs at monthly visits until alignment was completed. There was no statistically significant difference in the overall alignment duration between the two groups. The authors concluded that MOPs are no more effective in accelerating initial orthodontic alignment than conventional treatment. This study was performed for the initial alignment phase only.

## Discussion

There has been an increased interest in techniques to accelerate orthodontic tooth movement. The aim of this paper has been to present the different surgical techniques that have been developed to accelerate tooth movement and review the pertinent literature about these techniques.

One of the main reasons behind accelerating orthodontic tooth movement is to address the patients’ desire for shorter treatment time in orthodontic appliances.

All the techniques described above have decreased orthodontic treatment duration to some extent. Treatment duration was reduced by one-third to a quarter of the expected time with the PAOO surgery [18], and by half with piezocision surgery [45]. Rapid canine retraction [14] reduced the canine retraction phase, which can take an average of 6 months with routine appliance mechanics, to less than 1 month. The possibility of shortening the treatment time may be particularly advantageous for adult orthodontic patients, both in terms of accepting orthodontic treatment, and accepting multidisciplinary treatment plans, which may include pre-restorative orthodontics. When adult patients are more willing to go through orthodontic treatment to set the teeth in their correct positions with pre-restorative orthodontics, better restorative outcomes may be achieved.

The mechanism behind the accelerated tooth movement has been discussed by various authors. Initially, it was described as a “bony block movement” [10]; later, Wilcko and Wilcko [18] explained the acceleration of the tooth movement by the RAP, changing the previous understanding of the acceleration of the movement from a “bony block movement” to the physiological concept the “RAP”. Some of these procedures may also represent an alternative, albeit only in cases of mild skeletal discrepancies, to orthognathic surgery in patients who are not willing to undergo major procedures, by increasing the scope of tooth movement [41].

The effect of surgical techniques to accelerate orthodontic tooth movement on root resorption has been unclear. It was suggested that one of the advantages of corticotomy-assisted orthodontics is the protection from root resorption [28, 32, 39]. However, there have been contradictory findings on the effects of these techniques on root resorption [61, 66], especially in distinguishing the reason for the root resorption, i.e. whether root resorption is the effect of the procedures on root surface remodelling or related to the improper use of the surgery directly damaging the root.

To overcome the risk of damaging the tooth structure and compromising tooth vitality, the latest technological advancements can be used to create accurate surgical guides or can be used for navigation during the procedures. For each of the techniques, adequate preoperative imaging and planning is crucial to avoid any risks, especially when there is close root proximity. The majority of the publications on accelerated tooth movement using surgical techniques in the literature are predominantly case reports and case series, with only a limited number of controlled trials having been published. Although it is very difficult to make a direct comparison of the different techniques using the available data, the literature available sheds some light on the advantages and disadvantages of each procedure. Rapid canine retraction/dentoalveolar distraction [14, 36] only aimed to shorten a specific portion of the orthodontic treatment. This approach did not become popular over the years, due to its limited application—only for canine retraction—and the need for a special distractor device.

The PAOO, introduced by the Wilcko brothers, was shown to decrease the length of the orthodontic treatment up to 75% [29], while reducing unwanted effects, such as root resorption [32]. PAOO has a significant advantage which is the addition of bone augmentation during the decortications, which may prevent the formation of new fenestrations and bone dehiscences [27], and allows for the treatment of pre-existent ones, while thickening the alveolar housing [28]. The invasiveness of the surgical procedure is the major disadvantage of PAOO. Also, the length of the procedure and common postoperative complications, such as bruising and pain, make the acceptance of the procedure low both by the patients and clinicians.

Corticision [19] was introduced to use the RAP effect for accelerating the tooth movement without the invasiveness of the PAOO. The flapless approach decreases the surgical time, the postoperative discomfort, and the postoperative complications [19]. The main limitations of this procedure are the inability to graft and the trauma caused by the malleting. This technique did not seem to gain popularity over the years.

The Piezocision™ procedure, introduced by Dibart [22], combined the advantages of PAOO™, which is the possibility of grafting, and corticision, which is a flapless and thereby less invasive surgical procedure, and also added the benefits of using a piezoelectric knife instead of a bur for decortications. The surgical procedure is performed only on one side of the alveolus and the procedure can be performed in a very short time, especially compared to PAOO, with minimal patient discomfort and postoperative complications [22]. Healing, at the clinical level, is suggested to be more predictable and less painful [53]. The use of the piezoelectric knife ensures that only mineralized tissue is cut and produces precise osteotomies without osteonecrotic damage [23, 24]. Human studies [44, 45] confirmed that piezocision decreased total orthodontic treatment time. As this approach does not require flap elevation and a relatively simple procedure, patients are more likely to have the procedure done at different times for different parts of the jaw to meet specific mechanical requirements; therefore, a sequential approach is possible to make most of the RAP effect [41]. The latest technological advances make it possible to use surgical guides [42] or surgical navigation to increase the precision of the decortications and reduce the risk of damage to the roots. The possibility of scarring has been pointed out as a minor possible complication [47] of this technique, and suturing is recommended to avoid scarring; also, this should be discussed with the patient during the informed consent process. The acceptance of the piezocision procedure is shown to be considerably high compared to corticotomies [47]. It seems like as piezoelectric surgery is gaining more popularity amongst clinicians, piezocision has also been gaining popularity.

MOPs are the most recently introduced, relatively simple-to-use technique to accelerate tooth movement. It is a minimally invasive flapless approach, but does not allow for grafting, which is similar to corticision. The device used to make the MOPs is a simple handheld device, and there is no need for incisions. Careful planning of the location of the MOPs and the use of guides can help to avoid root damage, as this is a flapless approach. Patient discomfort and procedure time are much less compared to the other procedures previously described. The procedure may frequently require repeated applications, potentially adding to the cost and patient discomfort. In cases where the alveolar bone is thick and the effectiveness is a concern, other approaches such as PAOO™ or Piezocision™ should be considered for effective decortication.

A study published in 2014 evaluated clinician and patient interest in accelerated orthodontic treatment [67]. Close to 70% of the orthodontists were interested in adopting clinical procedures to reduce treatment time. Less invasive techniques had greater acceptability for both the clinician and the patients.

PAOO, Corticision, Piezocision, and MOPs (Propel) use different instruments (bur, mallet, piezoelectric knife, handheld screw device, etc.) for decortication. Dibart et al. [68] assessed and compared bone remodelling activity using a calvarial bone model, between burs, piezoelectric knives, and handheld screw devices, trying to answer the question whether all corticotomies affect the bone in the same way. They used 6 groups: (a) calvaria + media only (negative control); (b) piezotome sham injury, the piezotome with the BS1 insert was allowed to vibrate in media for five seconds with no injury; (c) piezotome injury; (d) bur “sham” injury, the bur mounted on high-speed handpiece span in media for 5 s with no injury; (e) bur injury; (f) injury by hand held screw perforation device.

A very surprising finding was that the sham piezotome group had high osteoclastic bone resorption, and osteoblastic differentiation and bone formation. This suggests that vibration may be producing a similar response to direct injury. The techniques induced varying degrees of bone remodelling. In the bone resorption model, the piezotome induced increased calcium release, TRAP activity, osteoclast differentiation, and bone resorption. Under bone formation conditions, the piezotome led to substantial levels of osteoblast differentiation with the formation of new osteoid far exceeding those observed with the bur and screw. The area of bone resorption and formation was greatest in the piezotome group, which extended to the controlateral calvarial side, possibly resulting from the combined effect of injury and vibration. The bur injury group had significant remodelling activity, which was limited to the injury region. The handheld screw device injury resulted in the lowest amount of osteoclastic and osteoblastic activity. The authors’ conclusion was that the piezotome had the greatest impact on bone resorption and formation. Their suggestion was that the high-frequency vibrations may intensify the response to injury.

A recent preliminary study by Kernitsky et al. [69] showed the importance of the depth of the corticotomies to accelerate the tooth movement and obtain the desired RAP effect. Previously, in 2017, Uribe et al. [70], published a randomized clinical trial to evaluate the efficiency of piezotome-corticision-assisted orthodontics in alleviating mandibular anterior crowding. They used 29 patients treated with piezotome-corticision or traditional orthodontics to correct mandibular crowding and found no difference in the treatment time required to correct the crowding. However, on close inspection of their methodology, it is mentioned that a modification of the piezocision was used and the depth of the cortical incision was kept limited to 1 mm; therefore, the expected RAP effect was not achieved. To determine if the depth of corticotomy undertaken with the piezoelectric knife could play a role in the intensity of the regional acceleratory phenomenon (RAP), Kernitsky et al. [69] carried out a preliminary study. They compared the effect of transcortical and intracortical penetration of the piezoelectric cuts and demonstrated that the intensity of the RAP in the rat is corticotomy depth dependent. They concluded that following a deep penetration with the piezoelectric knife, passing the cortex and reaching the medullary space, the RAP is more intense. This generates a higher level of osteoclastic activity and bone turnover, compared with a shallower injury. This, in turn, leads to more extensive bone demineralization, which may have unique clinical applications in terms of speed of tooth movement when combined with orthodontics. This is clinically relevant when decorticating the bone during surgically facilitated orthodontic procedures.

As the techniques to accelerate orthodontic tooth movement are gaining popularity, there is more research being published on these techniques. A recent case report published in 2020 by Hannequin et al. [71] presented the ortho-surgical treatment of a Class III malocclusion treated with corticotomies using a piezoelectric knife for accelerated presurgical decompensation and clear aligners, followed by mandibular sagittal split osteotomy. The clinical follow-up of aligner-mediated tooth movement, where aligners were changed every 4 days, was managed with a patient-managed smartphone application (dental monitoring) allowing early interception and correction of minute orthodontic movement errors. The authors demonstrated the good use of the dental monitoring technology when the accelerated rate of the tooth movement requires very close follow-up. Verna et al. [72] conducted a finite element study, which concluded that surgical interventions may influence the amount and type of tooth movement. They suggested that the transitory osteopenia generated by the injury to accelerate tooth movement would allow the shift of the centre of rotation of the movement more apically, favouring larger tooth movement for the corticotomized tooth, particularly for uncontrolled tipping. She et al. [73] also used finite element analysis to show the biomechanical effect of selective osteotomy and corticotomy on orthodontic molar uprighting and revealed how the different combinations of corticotomies had a biomechanical impact on orthodontic molar uprighting. They concluded the most extensive surgical approaches resulted in increased tooth movement, which supports Frost’s [15] description of the RAP where the stimulus magnitude determines the size of the affected region and the intensity of the response.

With all the surgical techniques to accelerate tooth movement, it is important to keep in mind that as the clinician can create a transient modification of the alveolus, more challenging movements may be achieved. Therefore, it is not only the speed of the treatment that potentially increases but also the possibility to successfully treat challenging adult patients [74].

It should be noted that the literature reviewed demonstrated an absence of consistency in terminology and set protocols. When planning future investigations these points should be considered, in addition to using long-term clinical trials in humans to obtain a clearer understanding of these techniques and the underlying biophysiology.

## Conclusions

Surgical techniques to accelerate tooth movements are not new. The journey of accelerated tooth movement that started as early as 1893 has been continuing with constant advancement, gaining popularity in the last decade, and the techniques have been evolving to reduce the disadvantages and improve the acceptability of treatment by patients and clinicians. Recently, there have been clinical modifications of corticotomies, with studies designed to compare the techniques where a flap was raised versus a flapless procedure, and a significant trend to do the decortication with a piezoelectric knife instead of a bur. Efforts to minimize the trauma to the patient and the chair time are also important points being considered in the development of new techniques. Long-term controlled clinical trials in humans are required to assess and compare the effectiveness of the different available techniques, their side effects, and long-term results.

Some conclusions may be drawn from the limited number of studies available:

- The surgical techniques reviewed and described in this article all *accelerate* orthodontic tooth movement at different rates. It is not possible to clearly compare the amount of reduction in treatment time achieved by these different techniques from the data available.
- Aside from the obvious ethical implications of animal experimentation, it is also difficult to see any clear benefit from such research. Future research should concentrate on human trials, the results of which will be directly applicable to human patients.
- Techniques to accelerate orthodontic tooth movement should be applied in selected cases when the benefits to the patient are clear. They should not be applied without clear indications for their benefits, which should always outweigh potential risks or harms.
- Some of the techniques described are gaining popularity (MOPs, piezocision), whereas others (rapid canine retraction, corticision) are not, due to limited applications, patient discomfort, or the invasiveness of the technique.
- Piezocision™ and PAOO™ have the advantage of *grafting* during the surgery, which allows for improvement of the hard and soft tissues and preventing periodontal defects which might occur due to a thin alveolar bone.
- Piezocision™, MOPs, and corticision are *flapless* approaches and are less invasive than PAOO™. Mallet use in corticision is traumatic for the patient. MOPs seem to be the least invasive application. PAOO™ appears to be the most invasive approach.
- The use of the *Piezoelectric knife* appears to have a greater impact on bone metabolism and creates a greater response to injury when compared to other techniques.
- The *depth of the injury* created during these procedures has a direct effect on the intensity of the RAP. Therefore, it plays a very important role in the effectiveness of the techniques.

## Data Availability

Not applicable.

## Abbreviations

MOPs: Micro-osteoperforations
PAOO: Periodontally accelerated osteogenic orthodontics
PDL: Periodontal ligament
RAP: Regional acceleratory phenomenon
TAD: Temporary anchorage device
